# Individual heterogeneity influences the effects of translocation on urban dispersal of an invasive reptile

**DOI:** 10.1186/s40462-022-00300-1

**Published:** 2022-01-15

**Authors:** Abigail B. Feuka, Melia G. Nafus, Amy A. Yackel Adams, Larissa L. Bailey, Mevin B. Hooten

**Affiliations:** 1 U.S. Department of Agriculture Animal and Plant Health Inspection Service, National Wildlife Research Center, 4101 Laporte Ave, Fort Collins, CO 80521-2154 USA; 2 U.S. Geological Survey, Fort Collins Science Center, 2150 Centre Avenue, Building C, Fort Collins, CO 80526-8118 USA; 3 Colorado State University, Department of Fish, Wildlife, and Conservation Biology and Graduate Degree Program in Ecology, Fort Collins, CO 80523-1474 USA; 4 The University of Texas at Austin, Department of Statistics and Data Sciences, Welch 5.216, 105 E 24th St D9800, Austin, TX 78705-1576 USA

**Keywords:** Bayesian hierarchical model, *Boiga irregularis*, Brown treesnake, Recursive computing, State space model, Translocation

## Abstract

**Background:**

Invasive reptiles pose a serious threat to global biodiversity, but early detection of individuals in an incipient population is often hindered by their cryptic nature, sporadic movements, and variation among individuals. Little is known about the mechanisms that affect the movement of these species, which limits our understanding of their dispersal. Our aim was to determine whether translocation or small-scale landscape features affect movement patterns of brown treesnakes (*Boiga irregularis*), a destructive invasive predator on the island of Guam.

**Methods:**

We conducted a field experiment to compare the movements of resident (control) snakes to those of snakes translocated from forests and urban areas into new urban habitats. We developed a Bayesian hierarchical model to analyze snake movement mechanisms and account for attributes unique to invasive reptiles by incorporating multiple behavioral states and individual heterogeneity in movement parameters.

**Results:**

We did not observe strong differences in mechanistic movement parameters (turning angle or step length) among experimental treatment groups. We found some evidence that translocated snakes from both forests and urban areas made longer movements than resident snakes, but variation among individuals within treatment groups weakened this effect. Snakes translocated from forests moved more frequently from pavement than those translocated from urban areas. Snakes translocated from urban areas moved less frequently from buildings than resident snakes. Resident snakes had high individual heterogeneity in movement probability.

**Conclusions:**

Our approach to modeling movement improved our understanding of invasive reptile dispersal by allowing us to examine the mechanisms that influence their movement. We also demonstrated the importance of accounting for individual heterogeneity in population-level analyses, especially when management goals involve eradication of an invasive species.

**Supplementary Information:**

The online version contains supplementary material available at 10.1186/s40462-022-00300-1.

## Background

Invasive species are among the top threats to global biodiversity [1]. Identifying invasion pathways and preventing translocation are the first lines of defense, but for individuals that evade these efforts, early detection and rapid response are considered the most efficient and cost-effective ways to prevent the spread of invasive species [2–4]. Early detection involves surveying for incipient invasive populations before they become established, and rapid response is the set of actions used to eradicate those populations [3]. The ability to understand and predict the dispersal of an invasive species through a novel environment allows managers to better target search and removal efforts.

An understanding of the mechanistic components of animal movement is critical to making informed predictions about invasive species dispersal. Nathan et al. (2008) outlined these components as the animal’s internal state (attributes internal to the animal such as stress levels, health, and behavior), how it moves, its navigation capacity, and external factors (those external to the animal such as weather, time of day, and landscape features) influencing its movement [5]. While species-level or population-level summaries of these mechanisms are useful for answering broad questions about movement, each of these mechanisms varies at the individual level, making individual heterogeneity an important consideration when making population-level movement inference [6–8]. This is especially true for invasive species, where the aberrant movements of a few individuals can ultimately dictate the speed of an invasion [9]. Variation in dispersal patterns also affects invader survival [10] and reproduction [11, 12], which has implications for population and metapopulation dynamics within an invasion [13, 14]. Eradication of an invasive species hinges on our ability to detect and remove all individuals, including those with particularly high movement propensity. Studies with the goal of informing eradication efforts of invasive species must consider individual heterogeneity to ensure coverage of all individuals within a population, allowing managers to determine how heavily they can rely on population-level averages of movement parameters. Thus, understanding how detectability changes with variation in a movement mechanism is useful in early detection and rapid response efforts [15]. We use the term “individual heterogeneity” in this work to refer to the differences in movement parameters among individuals in a population. Although this term can also be used to describe heterogeneity in a single individual’s movements over time, we focus on among-individual variation because the applications of this work involve the management of invasive reptile populations.

The cryptic appearance of many invasive reptiles makes them some of the most difficult invasive species to detect and remove [16]. Reptiles comprise 15% of all invasive species and have caused the decline, range contraction, and extinction of native species around the world, representing a serious ecological threat [17, 18]. Most reptile invasive ranges consist of warm tropical or subtropical climates. The relatively stable ambient temperatures and lack of defined breeding seasons, characteristic of these environments, allow reptiles to be active year-round in most of their invasive ranges. On smaller temporal scales, their feeding strategies range from active foraging (e.g., brown anoles, [19]) to ambush predation (e.g., Burmese pythons, [20]). Species that use the latter strategy may not move for days to weeks before and after a large meal [21, 22]. Low seasonal variation in climate and sporadic movement due to ambush predation can make invasive reptile movement unpredictable and difficult to model without an understanding of factors that influence movement mechanisms.

Reptiles also exhibit a high degree of individual heterogeneity that further complicates predicting their movements. For some species, heterogeneity within populations has been attributed to differences in personality and behavioral syndromes, such as aggression [23] or boldness [24], as opposed to passivity or shyness, respectively. Movement patterns can also change with the habitat an individual uses [25], its body condition [22], and whether an individual is located on the periphery of its invasive range versus in the core [26]. Further, behavior and landscape position may interact in situations where bold individuals make more frequent or longer movements through a low-quality habitat matrix than shy individuals [27]. These sources of individual heterogeneity are rarely incorporated as mechanisms in the movement process of a reptile (but see [28]). For example, quantifying the probability an individual moves from one habitat patch to another on a given day, and which habitat patch it will most likely move to, is invaluable to early detection and rapid response planning for cryptic species that have low detection probabilities. However, the landscape arrangement of a species’ invasive range may be vastly different from that of its native range, limiting the use of previous movement research to inform eradication efforts. An understanding of the mechanisms driving reptile movement in their invasive ranges can improve our ability to predict their spread or conduct an eradication program. There are multiple ways to account for this individual heterogeneity when modeling animal movement. Individual- (or agent-) based models allow for simulation of individual trajectories while allowing for complexity, such as changes in life history or intraspecific interactions [29, 30]. However, these simulations do not provide statistical inference on movement parameters or population-level structure. Bayesian hierarchical models allow for parameter estimation at both the individual and population levels, but are limited in their mechanistic realism by the amount of data needed to fit the model [31]. Approaches exist to merge these two methodologies [32, 33], and they are inherently related in that the data level of a hierarchical model is a simple individual-based movement model if it can generate a movement trajectory.

The brown treesnake (*Boiga irregularis*) invasion on the island of Guam is one example of a system where a mechanistic approach to movement inference is beneficial. The brown treesnake is a generalist predator responsible for the extirpation or severe decline of all of Guam’s native terrestrial vertebrates [34–36]. It is highly cryptic with very low detection probabilities during visual surveys [15]. Brown treesnakes use their cryptic coloring and body shape to aid nocturnal hunting, which involves a mixture of ambush predation and active foraging. They do not move for up to a week after consuming a large prey item [22, 35]. Within its native range, the brown treesnake has only two other snake predators and one only consumes its eggs, and it has no major predation pressure in its invasive range on Guam [37]. Like many invasive reptiles, a primary invasion pathway for brown treesnakes is the transportation system [35, 38]. However, little is known about how this species navigates through patchy landscapes, such as an urban port of entry, or how it behaves after translocation into a novel environment. Translocation is a source of stress for most animals, including snakes, and can cause changes in an animal’s stress hormone levels [39–41]. Translocation often involves a change in habitat, especially if a snake is arriving in an urban port of entry. An early detection and rapid response program exists for brown treesnakes [42], but the way in which brown treesnakes navigate urban landscapes or how this might be affected by translocation has not been quantified.

Brown treesnakes, being poikilothermic and utilizing both ambush and active hunting strategies, are expected to exhibit periods of high and low movement (hereafter, “movement” and “encamped” states) in a fragmented urban environment [36]. Morales et al. (2004) introduced the use of multi-state models to accommodate the effects of animal behavior on movement mechanisms [43], but these types of models are typically fit to mammalian or avian telemetry data because those species provide an abundance of relocations obtained through GPS or satellite telemetry devices. The high numbers of brown treesnakes on Guam provide a unique opportunity to obtain extensive reptilian telemetry data, allowing us to explore the mechanisms of invasive reptile movement within this system. We conducted a field experiment in which we translocated brown treesnakes into an urban area to simulate inter-island translocation into an urban port of entry, a common entry pathway for invasive species [3, 38]. We compared translocated snake movement parameters to those of snakes that were not translocated (resident snakes) to determine the effect of translocation on movement patterns. We translocated snakes from both forest and urban environments and compared them to non-translocated (resident) snakes in the same urban area to determine if there are differences in movement patterns depending on a snake’s habitat of origin.

We had three objectives for this study. First, our goal was to test if the probability of movement differs with translocation or snake origin. We hypothesized that translocated and resident snakes would have a similar probability of movement, as demonstrated by previous studies [44–46]. Second, we wanted to test if the probability of movement depended on small-scale landscape features (i.e., external state change). We predicted snakes located in natural refugia such as trees and grass would be less likely to move than snakes utilizing a building or paved surface where we assumed there to be lower diversity in microclimates to select for thermoregulation [47, 48]. We predicted this effect would be more pronounced in snakes translocated from forests, where we assumed they did not have prior experience using buildings or paved surfaces, and thus were less likely to use them as refugia. Finally, we wanted to test if movement parameters (e.g., step length, turning angle) differ between translocated versus non-translocated snakes and between translocated snakes from forests versus urban areas. We predicted translocated snakes would make longer, more unidirectional movements than resident snakes based on previous snake translocation research [44, 45, 49, 50] and that this effect would be stronger in snakes translocated from forests. We developed a Bayesian hierarchical model to account for individual heterogeneity in snake behavior while using treatment-level parameters to test these hypotheses regarding the effect of translocation and small-scale landscape features on brown treesnake movement.

## Methods

### Study site

This study was conducted on Andersen Air Force Base in Yigo, Guam, USA. We used the Andersen Air Force Base Housing Unit as our urban study site to emulate features typical of an urban port of entry such as paved roads, buildings, patchy tree cover, and short ground vegetation. Specifically, this 230 ha area was characterized by single trees or patches of trees within a matrix of short-cut (<2 cm) lawn with a network of paved roads, houses, and a golf course. Dominant tree species included *Cocos nucifera* (niyok or coconut palm), *Delonix regia* (arbol del fuego or flame tree), and *Casuarina equisetifolia* (gago or Australian pine). Our study site abutted contiguous forest on the eastern edge and a mix of suburban homes interspersed with smaller forest patches on all other sides.

### Experimental design and data collection

We collected brown treesnakes by hand during nighttime visual surveys and with mouse-baited traps that were checked daily. We limited our study to small snakes between 700 and 1000 mm snout-to-vent length because snakes of this size are most often found in Guam’s transit system and thus at highest risk of translocation [38]. We assigned snakes to three treatment groups based on origin and translocation status: Resident urban (hereafter, “resident”), translocated from a forest site to our urban site (hereafter, “forest to urban”), and translocated from a different urban area to our study site (hereafter, “urban to urban”). We chose these groups to determine how snakes translocated into an urban area (simulating an urban port of entry) move compared to snakes that normally reside in an urban area. We translocated snakes from both forests and other urban areas to determine if habitat of origin changes how translocated snakes move in a new environment. For example, we expected snakes from other urban areas to exhibit a less pronounced effect of translocation of their movements, because they presumably have more experience navigating a landscape with pavement and buildings.

We found resident snakes in our urban study site, equipped them with a transmitter in the laboratory and returned them to the location in which they were found the following day. To simulate travel by ship, we collected translocated snakes from forests and urban areas around Guam, held them in the laboratory for up to six days with access to water and relocated them into our urban site. Though all snakes likely experienced increased stress from capture, handling, and tagging, we considered the longer holding time of translocated snakes to be an increase in stress relative to residents. We did not have a large enough sample size to test the effects of holding time on movement. We obtained translocated snakes from locations that were at least 2 square kilometers of contiguous tree cover or human development (assessed visually using satellite imagery), respectively for forest to urban and urban to urban snakes, and 5 kilometers away from the boundary of our study site. All snakes were equipped with a very-high frequency (VHF) transmitter (Holohil BD-2, 1.4–1.6 g, Holohil Systems Ltd., Carp, Ontario, Canada) attached to their tail using the subdermal stitch attachment method described by Riley et al. [51], with an additional wrap of medical tape around the transmitter to reduce the risk of catching on vegetation. We ensured the mass of the transmitters did not exceed 5% of the snake’s body mass.

We tracked one cohort of brown treesnakes in each of the following seasons: Two wet seasons (June to September 2018 and 2019) and one dry season (February to April 2019), with snakes in all three experimental treatment groups in each season. We originally incorporated these seasonal effects due to hypothesized decreases in movement during the dry season, but ultimately combined them due to small treatment group sample sizes in each season. We tracked snakes daily between 0800 and 1400 h primarily via homing and triangulated their location using a minimum of five azimuths when we could not access a snake’s daytime location. Brown treesnakes primarily move at night, and we assumed snakes were stationary during our daytime tracking and thus this analysis quantifies linear nightly movement distance, discounting smaller-scale nightly movements that result in individuals returning to the same daytime refugia. Because our goal was to evaluate movement in the context of early detection and rapid response programs, we felt focusing on this scale of movement was justifiable for our objectives. Turning angles and step lengths thus describe summaries of daily dispersal of snakes rather than fine-scale, or step-by-step, movements.

Snakes were often underground, in tree branches, or otherwise hidden from view and thus the risk of an observer disturbing a snake was low. We recorded homed snake positions using Garmin® GPSMAP 64x (Garmin International, Inc., Olathe, Kansas, USA) series global positioning units, with signal error primarily ranging from 3 to 5 m, and occasionally ranging up to 15 m of error in very few cases. Posterior mode positions from triangulation data were estimated using the R package “razimuth,” which fits a Bayesian azimuthal telemetry model to estimate the individual’s location [52, 53]. We validated the accuracy of our triangulation relocations by triangulating and homing a subset of snakes, and found the median difference in the homing estimate and triangulation posterior mode was 6 m, and because the spatial extent of our landscape features was much larger than this (see *Landscape Covariates* below), we felt use of these triangulated estimates was appropriate.

### Landscape covariates

To match an appropriate scale of snake movement, we used 0.7-m resolution satellite imagery (Satellite Imaging Corporation, KOMPSAT-3 satellite sensor) to derive landscape covariate data in our study area. The imagery was collected September 24, 2018, at the end of the first season of data collection, and no major landscape changes occurred during the course of our study. To classify the landscape into categories, we created a training dataset of 100 digitized polygons each of pavement, building, grass, and tree cover within our study area that we used to train a random forest algorithm via R package “randomForest” [54] to classify pixels of a raster image. As with all satellite imagery and classification algorithms, there is inevitable error in classification accuracy. However, because we used high-resolution satellite imagery in our random forest algorithm, we were able to distinguish the shapes of trees, buildings, and roads from our classified image and verify their alignment with the original imagery. A variogram analysis indicated the effective spatial extent of our raster image was over 1000 m. While our data are fine-scale to allow for more precise feature definitions, the variogram analysis suggests that landscape features in our study site are typically not small. Thus, most of our landscape features are not sub-meter in size, but our fine-scale data allows for more precise estimates of feature boundaries in our study area.

### Model specification

Our model consists of data-level, individual-level process, and experimental treatment-level process sub-models. We explain the model in terms of these sub-models, and we list the full model in Additional File 1. In our data model, we converted daily-resolution relocations $\varvec{s}_t$ into the difference of each successive pair, denoted by the $2\times 1$ vector $\varvec{\delta }_t$. This vector represents the change in position between a snake’s location on day $t-1$ and day *t*. Because snakes do not move every day, we modeled velocity to arise from a mixture of normal distributions that represent two behavioral states: movement and encamped [43]. The mixture probability $p_{it}$ is the nightly probability of movement for individual *i*, or probability an individual makes long movements with potentially directed turning angles, and $1-p_{it}$ is the probability of encampment, or an individual making short movements with random turning angles. We specified the data model as

1 $$\begin{aligned} \begin{aligned} \varvec{\delta }_{it}&= \varvec{s}_{it}-\varvec{s}_{it-1},\quad \text {for } t=3,...,T_i \text { and } i=1,...,N_j,\\ \varvec{\delta }_{it}&\sim {\left\{ \begin{array}{ll} \text {N}(\gamma _{i}{} \mathbf{M} (\theta _{i})\varvec{\delta }_{it-1},\sigma ^2_{1,i}{} \mathbf{I} ), &{} \text{ with } \text{ probability } p_{it}, \\ \text {N}(\varvec{0},\sigma ^2_{0}{} \mathbf{I} ), &{} \text{ with } \text{ probability } 1-p_{it}, \end{array}\right. } \\ p_{it}&= \Phi (\varvec{x}'_{it-1}\varvec{\beta }_{i}), \end{aligned} \end{aligned}$$

where *t* is a timestep (night) for snake *i* in treatment group *j*. When a snake was in the movement state, its movement at time *t*, $\varvec{\delta }_{it}$, was auto-regressed on its last movement $\varvec{\delta }_{it-1}$. This previous night’s movement was multiplied by the propagation matrix $\mathbf{M}$, which rotates the direction of $\varvec{\delta }_{it}$ using

2 $$\begin{aligned} \begin{aligned} \mathbf{M} \equiv \begin{pmatrix} \cos (\theta _i) &{} -\sin (\theta _i) \\ \sin (\theta _i) &{} \cos (\theta _i) \end{pmatrix}, \end{aligned} \end{aligned}$$

in which $\theta _i$ controls the turning angle (in radians) of snake *i*. To create more realistic trajectories, we multiplied the autoregression coefficient $\gamma _i$ to each snake’s propagation matrix to dampen its effect. Values of $\gamma _i$ near one create strongly patterned, repetitive trajectories, because there is less variation in the turning angle between one movement and the next. Values of $\gamma _i$ near zero lessen the adherence to the previous movement’s pattern, allowing for more variation in turning angles. For example, if an individual had values $\gamma _i=1$ and $\theta _i=\pi /2$, it would always make 90-degree turns. If $\gamma _i=0.5$ and $\theta _i=\pi /2$, the individual would make 90-degree turns some of the time, but it could make turns more or less than 90 degrees as well.

The variance parameters, $\sigma ^2_0$ and $\sigma ^2_{1,i}$ multiplied by identity matrix $\mathbf{I}$, control the variation in step length and direction for each state, where twice the standard deviation accounts for 95% of the movement kernel at each step. Lower values of these parameters create trajectories where the individual’s steps will be approximately the same length. We specified the encamped state to have 95% movement kernel of 10 m ($\sigma _0=5$) for all snakes, which is typical tree canopy diameter at our study site plus 5 m of GPS error, or the maximum distance we expected a snake’s position to change when it is resting. The short step lengths in the encamped state were combined with random turning angles (mean $=\varvec{0}$) to model homing behavior, allowing the snake to move around the canopy of the same tree from day to day, behavior we still considered “encamped”.

The movement state kernel size, dictated by $\sigma ^2_{1,i}$, varied for each individual. Preliminary models fit to individuals with no movements greater than 10 m could not differentiate between the two states and tended to estimate $\sigma ^2_{1,i}<\sigma ^2_0$. For these individuals, we manually set all steps to be in the encamped state to ensure all parameters were identifiable. We also let $\theta _i$ vary among individuals, allowing for differences in turning angles among snakes.

To incorporate the potential of external effects on the snake movement process, we specified movement probability $p_{it}$ as a probit-linear function of the small-scale landscape feature a snake was using at the starting position of each movement, $\varvec{x}_{it-1}$, specifically trees, grass, pavement, or buildings, as classified from satellite imagery. We allowed each individual to have its own vector of regression coefficients $\varvec{\beta }_i$, because we expected individual heterogeneity in this relationship within the treatment groups.

Each individual snake had its own set of movement parameters ($\gamma _i$, $\theta _i$, and $\sigma ^2_{1,i}$) and parameters dictating the probability of movement ($\varvec{\beta }_i$). Our individual-level process model was comprised of these parameters, which arose from normal distributions that are parameterized by treatment-level means and variances, indexed by *j*:

3 $$\begin{aligned} \begin{aligned} \varvec{\beta }_{i}&\sim \text {N}(\varvec{\mu }_{\beta ,j}, \varvec{\Sigma }_{\beta ,j}),\quad \text {for } j=1,...,3,\\ \text {logit}(\gamma _{i})&\sim \text {N}(\mu _{\text {logit}(\gamma ),j}, \sigma ^2_{\text {logit}(\gamma ),j}),\\ \theta _{i}&\sim \text {N}(\mu _{\theta ,j},\sigma ^2_{\theta ,j}),\\ \text {log}(\sigma _{1,i})&\sim \text {N}(\mu _{\text {log}(\sigma _1),j},\sigma ^2_{\text {log}(\sigma _1),j}). \end{aligned} \end{aligned}$$

Though $\theta _i$ does not have normal support, the normal distribution with hyperpriors that place the majority of its mass between 0 and $2\pi$ produced better mixed Markov chain Monte Carlo (MCMC) chains than the wrapped Cauchy distribution, a circular distribution traditionally used for turning angle parameters.

Treatment-level means arose from normal distributions, variance parameters arose from inverse gamma distributions, and the covariance matrix for the vector $\varvec{\beta }_i$ arose from an inverse Wishart distribution:

4 $$\begin{aligned} \begin{aligned} \varvec{\mu }_{\beta ,j}&\sim \text {N}(\varvec{\mu }_{\beta ,\text {pop}},\varvec{\Sigma }_{\beta ,\text {pop}}),\\ \mu _{\text {logit}(\gamma ),j}&\sim \text {N}(\mu _{\text {logit}(\gamma ),\text {pop}},\sigma ^2_{\text {logit}(\gamma ),\text {pop}}),\\ \mu _{\theta ,j}&\sim \text {N}(\mu _{\theta ,\text {pop}},\sigma ^2_{\theta ,\text {pop}}),\\ \mu _{\text {log}(\sigma _{1,j})}&\sim \text {N}(\mu _{\text {log}(\sigma _1),\text {pop}},\sigma ^2_{\text {log}(\sigma _1),\text {pop}}),\\ \varvec{\Sigma }_{\beta ,j}^{-1}&\sim \text {Wish}((\mathbf{S} \nu )^{-1},\nu ),\\ \sigma ^2_{\text {logit}(\gamma ),j}&\sim \text {IG}(q_{\text {logit}(\gamma ),\text {pop}},r_{\text {logit}(\gamma ),\text {pop}}),\\ \sigma ^2_{\theta ,j}&\sim \text {IG}(q_{\theta ,\text {pop}},r_{\theta ,\text {pop}}),\\ \sigma ^2_{\text {log}(\sigma _{1},j)}&\sim \text {IG}(q_{\text {log}(\sigma _1),\text {pop}},r_{\text {log}(\sigma _1),\text {pop}}). \end{aligned} \end{aligned}$$

The treatment-level means were used for testing our hypotheses at the treatment scale. We analyzed heterogeneity among individuals in each treatment group as a derived quantity by calculating the sample variance of all individual-level estimates of each parameter.

### Proposal recursive computation

We used the two-stage model-fitting technique described by McCaslin et al. [55], which allowed us to fit individual first-stage models to each snake in parallel using package “parallel” in R version 3.6.1 [56] and then use those samples as proposals in the second stage to fit the full hierarchical model [57, 58].

We fit the first-stage models using a latent variable parameterization, where $z_{t}$ is equal to one when a snake is in the movement state at time *t* and $z_{t}$ is equal to zero when a snake is in the encamped state at time *t*. We used an additional latent variable $v_t$ to induce conjugate updates on $\varvec{\beta }_i$, as described by Albert and Chib [59]. We fit the following model to each snake’s trajectory in the first stage:

5 $$\begin{aligned} \begin{aligned} \varvec{\delta }_{t}&= \varvec{s}_{t}-\varvec{s}_{t-1}\quad \text {for } t=3,...,T,\\ \varvec{\delta }_{t}&\sim {\left\{ \begin{array}{ll} \text {N}(\gamma _{}{} \mathbf{M} (\theta )\varvec{\delta }_{t-1},\sigma ^2_{1}{} \mathbf{I} ), &{} z_{t}=1, \\ \text {N}(\varvec{0},\sigma ^2_0 \mathbf{I} ), &{} z_{t}=0, \end{array}\right. }\\ z_{t}&= {\left\{ \begin{array}{ll} 0, &{} v_t \le 0,\\ 1, &{} v_t>0, \end{array}\right. }\\ v_t&\sim \text {N}(\varvec{x}'_{t}\varvec{\beta },1),\\ \varvec{\beta }&\sim \text {N}(\varvec{\mu }_{\beta }, \sigma ^2_{\beta }{} \mathbf{I} ),\\ \gamma&\sim \text {Beta}(\alpha _1,\alpha _2),\\ \theta&\sim \text {WC}(\mu _{\theta },\rho _{\theta }),\\ \sigma _{1}^2&\sim \text {IG}(q_1,r_1).\\ \end{aligned} \end{aligned}$$

When we integrate over latent variables $\mathbf{z}$ and $\mathbf{v}$, the probability of being in the movement state at time *t* is $p_t$=$\Phi (\varvec{x_t}'\varvec{\beta })$. Note the prior distribution for $\gamma$ is changed to beta, $\theta$ is changed to wrapped Cauchy, and $\sigma ^2_1$ is changed to inverse gamma (Eq. 5). This was to create first-stage MCMC parameter updates that match the support of each parameter and did not require tuning or supervision, because we used the prior as a proposal for the Metropolis-Hastings update on $\gamma$ and $\theta$ and a Gibbs update for $\sigma ^2_1$ in the first stage. We accounted for this difference in model specification by using a change of variables correction in the second stage Metropolis-Hastings updates [55].

After fitting the model, we obtained inference for back-transformed parameters $\varvec{\mu }_{\gamma }$, $\varvec{\mu }_{\sigma }$, and $\mathbf{p}$ using Monte Carlo integration [60].

### Priors

We specified the following temporary hyperpriors for the first stage individual models:

6 $$\begin{aligned} \begin{aligned} \varvec{\beta }&\sim \text {N}(\varvec{\mu }_{\beta }=\varvec{0}, \sigma ^2_{\beta }{} \mathbf{I} =1\cdot \mathbf{I} ),\\ \gamma&\sim \text {Beta}(\alpha _1=1,\alpha _2=1),\\ \theta&\sim \text {WC}(\mu _{\theta }=0,\rho _{\theta }=0.1),\\ \sigma _{1}^2&\sim \text {IG}(q_1=1,r_1=0.1),\\ \end{aligned} \end{aligned}$$

where **I** is the identity matrix. We specified the following priors for the full model:

7 $$\begin{aligned} \begin{aligned} \varvec{\mu }_{\beta ,j}&\sim \text {N}(\varvec{\mu }_{\beta ,\text {pop}}=\varvec{0},\sigma ^2_{\beta ,\text {pop}}{} \mathbf{I} =1\cdot \mathbf{I} ),\\ \mu _{\text {logit}(\gamma ),j}&\sim \text {N}(\mu _{\text {logit}(\gamma ),\text {pop}}=\text {logit}(0.7),\sigma ^2_{\text {logit}(\gamma ),\text {pop}}=1.5),\\ \mu _{\theta ,j}&\sim \text {N}\left( \mu _{\theta ,\text {pop}}=\pi ,\rho _{\theta ,\text {pop}}=\frac{\pi ^2}{4}\right) ,\\ \mu _{\text {log}(\sigma _{1,j})}&\sim \text {N}\left( \mu _{\text {log}(\sigma _1),pop}=\text {log}(20),\sigma ^2_{\text {log}(\sigma _1),\text {pop})}=100\right) ,\\ \sigma ^2_{\beta ,j}&\sim \text {IG}(q_{\beta ,\text {pop}}=0.01,r_{\beta ,\text {pop}}=10),\\ \sigma ^2_{\text {logit}(\gamma ,j)}&\sim \text {IG}(q_{\gamma ,\text {pop}}=0.001,r_{\gamma ,\text {pop}}=1000),\\ \sigma ^2_{\theta ,j}&\sim \text {IG}(q_{\theta }=2.5,r_{\theta }=0.5),\\ \sigma ^2_{\text {log}(\sigma _{1},j)}&\sim \text {IG}(q_{\text {log}(\sigma _1),\text {pop}}=0.001,r_{\text {log}(\sigma _1),\text {pop}}=1000).\\ \end{aligned} \end{aligned}$$

We used semi-informative prior distributions to minimize label switching and to ensure biological realism in our estimates. We used prior means of $\varvec{0}$ for the $\varvec{\beta }$ coefficients, and we used a prior covariance of one multiplied by the identity matrix **I**, which induced a diffuse distribution on the probit scale with no assumptions of the influence of covariates on movement probability or covariance between coefficients. The priors for $\mu _{\text {logit}(\gamma ),j}$ regularized the treatment means for $\text {logit}(\gamma )$ away from zero to prevent the movement state mean from being equal to $\varvec{0}$ and thus differentiated the movement and encamped states. The hyperparameters for $\mu _{\theta ,j}$ allow the normal distribution to be centered on $\pi$ with a variance that bounds the majority of the probability mass between 0 and $2\pi$. The mean for the prior distribution of $\mu _{\text {log}(\sigma ),j}$ was set to be the log of 20, where $2\times 20$ creates a 95% movement kernel of 40 m, which is the average distance small brown treesnakes typically move in a night [61]. We set the prior variance for $\mu _{\text {log}(\sigma ),j}$ to 100 to keep movement distances biologically realistic. We chose hyperparameters for treatment-level variance parameters to be relatively diffuse while keeping variance estimates within reasonable bounds of their respective transformations.

### Model fitting

We fit our hierarchical model using custom MCMC algorithms written in R version 3.6.1 [56]. We ran the first stage algorithms for 100,000 iterations each, the second stage algorithm for 50,000 iterations, and verified convergence and mixing by visually inspecting trace plots. We removed the first 10% of the second-stage iterations as burn-in. In the two-stage proposal-recursive method, all first-stage parameters are autocorrelated, and we would thus traditionally update all individual-level parameters jointly in the second stage. However, updating the large number of individual-level parameters jointly in the second stage may result in poor mixing, so we sampled each parameter from its first stage sample individually using the following generalized Metropolis-Hastings ratio:

8 $$\begin{aligned} r^{k}_{jl}=\frac{ \left[ \varvec{y}_i|\alpha ^{*}_{il},\alpha ^{k}_{i,1:l-1},\alpha ^{k-1}_{i,l+1:L}\right] \left[ \alpha ^{*}_{il}|\varvec{\psi },\alpha ^{k}_{i,1:l-1},\alpha ^{k-1}_{i,l+1:L}\right] \left[ \alpha _{il}^{k-1} | \mathbf {y}_i\right] _{*}}{\left[ \varvec{y}_i|\alpha ^{k-1}_{il},\alpha ^{k}_{i,1:l-1},\alpha ^{k-1}_{i,l+1:L}\right] \left[ \alpha ^{k-1}_{il}|\varvec{\psi },\alpha ^{k}_{i,1:l-1},\alpha ^{k-1}_{i,l+1:L}\right] \left[ \alpha _{il}^{*} | \mathbf {y}_i\right] _{*}}. \end{aligned}$$

where ${\alpha }_{il}$ is the $l{\text {th}}$ parameter in the vector of parameters for individual *i*. Superscript *k* indicates the index for the current MCMC iteration, $*$ indicates a proposed value, $\varvec{\psi }$ is a vector of hyperparameters for $\alpha _{il}$, and $_{*}$ indicates the second-stage proposal distribution (not the second-stage marginal posterior distribution of $\alpha _{il}$).

Updating each of the correlated $\beta _{il}$ coefficients individually required the use of the joint distribution:

9 $$\begin{aligned} \begin{pmatrix} \varvec{\beta }_o\\ \varvec{\beta }_u \end{pmatrix} \sim \text {N} \left( \begin{pmatrix} \varvec{\mu }_{\varvec{\beta },o}\\ \varvec{\mu }_{\varvec{\beta },u} \end{pmatrix}, \begin{pmatrix} \varvec{\Sigma }_{\varvec{\beta },oo}, \varvec{\Sigma }_{\varvec{\beta },ou}\\ \varvec{\Sigma }_{\varvec{\beta },uo} \varvec{\Sigma }_{\varvec{\beta },uu} \end{pmatrix} \right) \end{aligned}$$

where *o* indicates observed, or updated, $\beta$ coefficients, and *u* indicates unobserved, or coefficients not yet updated in the MCMC iteration. In our model, this creates the following Metropolis-Hastings ratio used to update each $\beta _i$ coefficient, assuming $\gamma _i$, $\theta _i$, and $\sigma ^2_{1,i}$ have already been updated:

10 $$\begin{aligned} r^{k}_{jl}=\frac{ \begin{matrix} &{}\left( \prod _{t=3}^{T_i}\left[ \varvec{\delta }_{ti}|\gamma ^{k}_i,\theta ^{k}_i,\sigma ^{2(k)}_{1,i},\beta ^{*}_{il},\beta ^{k}_{i,1:l-1},\beta ^{k-1}_{i,l+1:L}\right] \right) \times \\ &{}\left[ \beta ^{*}_{il}|\varvec{\mu }_{\beta ,j},\varvec{\Sigma }_{\beta ,j},\beta ^{k}_{i,1:l-1},\beta ^{k-1}_{i,l+1:L}\right] \left[ \beta _{il}^{k-1} | \varvec{\delta }_{i}\right] _{*} \end{matrix} }{\begin{matrix} &{}\left( \prod _{t=3}^{T_i}\left[ \varvec{\delta }_{ti}|\gamma ^{k}_i,\theta ^{k}_i,\sigma ^{2(k)}_{1,i},\beta ^{k-1}_{il},\beta ^{k}_{i,1:l-1},\beta ^{k-1}_{i,l+1:L}\right] \right) \times \\ &{}\left[ \beta ^{k-1}_{il}|\varvec{\mu }_{\beta ,j},\varvec{\Sigma }_{\beta ,j},\beta ^{k}_{i,1:l-1},\beta ^{k-1}_{i,l+1:L}\right] \left[ \beta _{il}^{*} | \varvec{\delta }_{i}\right] _{*} \end{matrix}}. \end{aligned}$$

Our movement model parameters $\gamma$, $\theta$, and $\sigma ^2_1$, were not sampled jointly in the first stage, thus the process distribution $\left[ \alpha _{il}|\varvec{\psi },\alpha _{i,1:l-1},\alpha _{i,l+1:L}\right]$ in Eq. 8 simplified to $\left[ \alpha _{il}|\varvec{\psi }\right]$ yielding

11 $$\begin{aligned} \begin{aligned} r^{k}_{jl}=\frac{ \left[ \varvec{y}_i|\alpha ^{*}_{il},\alpha ^{k}_{i,1:l-1},\alpha ^{k-1}_{i,l+1:L}\right] \left[ \alpha ^{*}_{il}|\varvec{\psi }\right] \left[ \alpha _{il}^{k-1} | \mathbf {y}_i\right] _{*}}{\left[ \varvec{y}_i|\alpha ^{k-1}_{il},\alpha ^{k}_{i,1:l-1},\alpha ^{k-1}_{i,l+1:L}\right] \left[ \alpha ^{k-1}_{il}|\varvec{\psi }\right] \left[ \alpha _{il}^{*} | \mathbf {y}_i\right] _{*}}. \end{aligned} \end{aligned}$$

for each of $\gamma$, $\theta$, and $\sigma ^2_1$.

To reduce computation time, we approximated $\left[ \alpha _{il} | \mathbf {y}_i\right] _{*}$ using the kernel density estimate calculated from each parameter’s first-stage samples. We evaluated kernel densities using the R package “ks” [62]. See Additional File 2 for a more detailed explanation of the Metropolis-Hastings ratios used in our model.

### Hypothesis testing

We assessed the differences in treatment-level means for all movement parameters and movement probabilities by calculating the difference between pairs of parameters as a derived quantity and using the posterior mean of that quantity for hypothesis testing. We also used posterior probability to assess the strength of the effect, which we calculated as the proportion of times a treatment group’s parameter estimate was larger than another treatment group’s parameter estimate over all MCMC iterations [63].

## Results

We used locations from 84 brown treesnakes across the three treatment groups and three seasons: 29 in the resident group, 32 in the forest-to-urban group, and 23 in the urban-to-urban group. Table 1 lists sample sizes of snakes tracked in each season by treatment group and cumulative number of relocations. Individual trajectories ranged from 5 to 77 relocations. Snakes ranged in size from 706 to 991 mm snout-to-vent length and ranged in weight from 28 to 118 g. A total of 10 snakes moved outside of the study site into the more contiguous abutting forest: three of which were resident snakes, five snakes translocated from forests, and two translocated from other urban areas. Details on these snakes are included in Additional File 3: Table S1.

**Table 1:** Sample size of brown treesnakes tracked in each of three seasons: wet season 2018, dry season 2019, and wet season 2019

Season	Treatment	Number of individuals	Number male	Number female	Number of relocations
Wet 2018	Resident	10	5	5	521
Wet 2018	Forest to Urban	10	4	6	424
Wet 2018	Urban to Urban	3	1	2	107
Dry 2019	Resident	10	3	7	411
Dry 2019	Forest to Urban	10	3	7	353
Dry 2019	Urban to Urban	8	1	7	265
Wet 2019	Resident	9	3	6	425
Wet 2019	Forest to Urban	12	5	7	329
Wet 2019	Urban to Urban	12	8	4	315

Resident snakes were non-translocated snakes in an urban area, forest to urban snakes were translocated from a forest to an urban area, and urban to urban snakes were translocated from an urban to a novel urban area

### Probability of movement

**Fig. 1:**
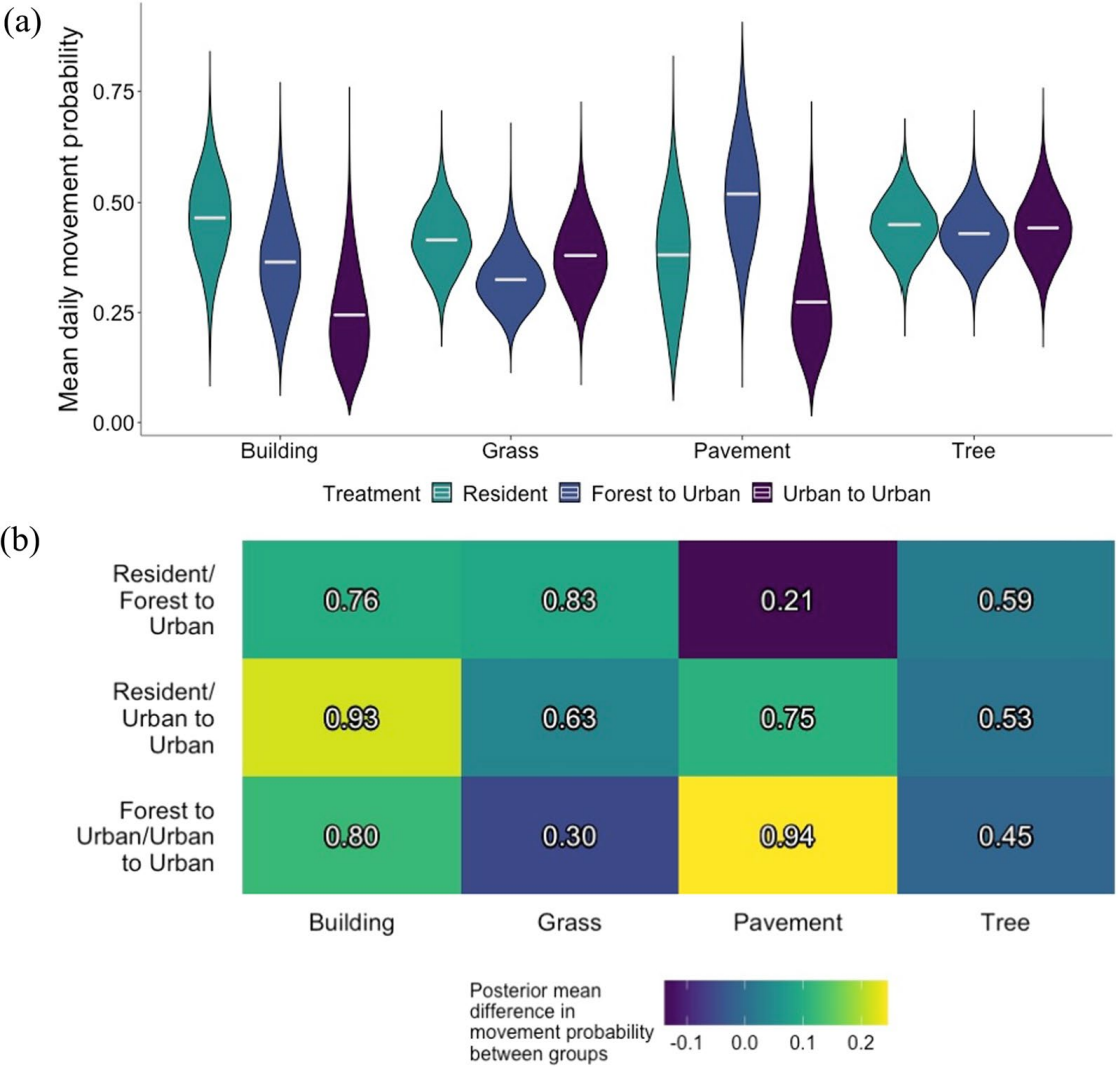
**a** Marginal posterior distributions for mean probabilities of brown treesnake movement by treatment group when a snake was located in each listed landscape feature $\mu _{p_{t}}$. Resident snakes were non-translocated snakes in an urban area, forest to urban snakes were translocated from a forest to an urban area, and urban to urban snakes were translocated from an urban to a novel urban area. **b** Heat map of marginal posterior mean difference in brown treesnake movement probability between treatment groups. The colors represent the difference in the first listed treatment group’s posterior mean compared to the second listed treatment group’s posterior mean. The posterior probability that the first listed treatment group is greater than the second listed treatment group is printed in each cell

The average nightly probability of a brown treesnake being in the movement state (i.e., movement probability) was low overall, with treatment group posterior means ranging from 0.24 to 0.52 (Fig. 1a). Posterior probabilities indicate most differences in movement probability among translocation treatment groups were weak (Fig. 1b). The two exceptions were when snakes used urban landscape features. When in buildings, resident snakes had a posterior mean movement probability 0.22 higher than snakes translocated from urban areas with a posterior probability of 0.93 (Fig. 1b). When on pavement, snakes translocated from forests had the largest movement probability, with a posterior mean movement probability of 0.52 (95% CI: 0.30, 0.75). This was 0.25 greater than the posterior mean for snakes translocated from urban areas with a posterior probability of 0.94 (Fig. 1b), suggesting snakes translocated from forests were less likely to settle on paved surfaces than snakes translocated from urban areas.

Within treatment groups, we found few differences in brown treesnake movement probabilities among small-scale landscape features. The two exceptions involved translocated snakes moving from landscape features that were uncommon in their location of origin. Specifically, when snakes translocated from urban areas were in trees, their posterior mean movement probability was 0.20 greater than in buildings with posterior probability of 0.93 (Table 2). When forest to urban snakes were moving from pavement, their posterior mean probability of movement was 0.19 greater than when on grass with posterior probability of 0.93 (Table 2).

**Table 2:** Posterior mean effect size in treatment-level probability of brown treesnake movement from small-scale landscape features

Comparison of marginal posterior distributions of movement probability from landscape features	Effect size of comparison (posterior probability)		
	Resident	Forest to Urban	Urban to Urban
Tree > Pavement	0.07 (0.70)	− 0.09 (0.25)	**0.17 (0.90)**
Tree > Grass	0.03 (0.68)	0.10 (0.87)	0.06 (0.73)
Tree > Building	− 0.02 (0.43)	0.06 (0.70)	**0.20 (0.93)**
Pavement > Grass	− 0.03 (0.41)	**0.19 (0.93)**	− 0.11(0.21)
Pavement > Building	− 0.08 (0.29)	0.15 (0.83)	0.03 (0.58)
Grass > Building	− 0.05 (0.31)	− 0.04 (0.37)	0.13 (0.85)

Posterior probabilities of differences are listed in parentheses. Resident snakes were non-translocated snakes in an urban area, forest to urban snakes were translocated from a forest to an urban area, and urban to urban snakes were translocated from an urban to a novel urban area. Values associated with posterior probabilities $\le$0.1 or $\ge$0.9 are in bold

**Fig. 2:**
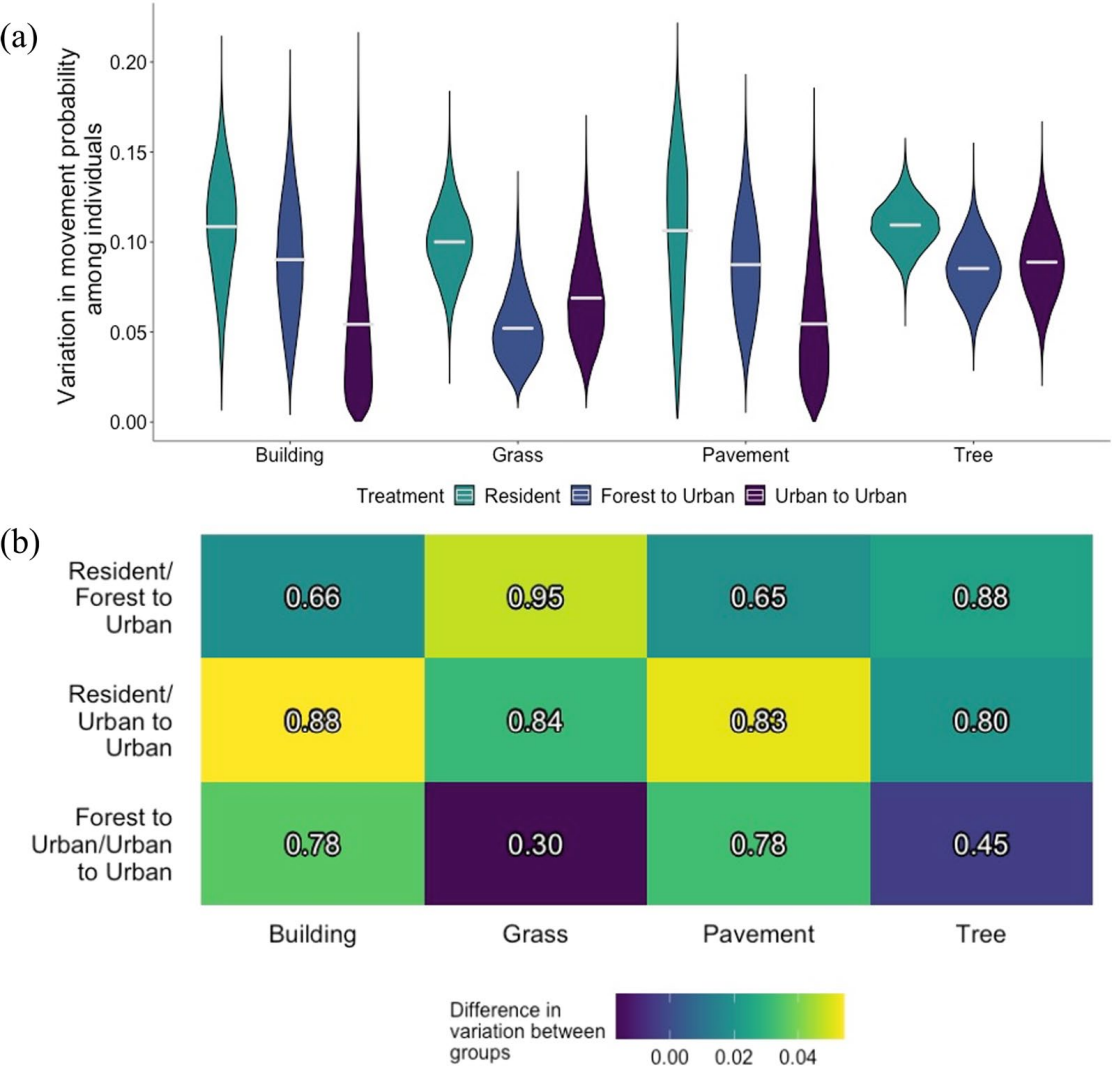
**a** Marginal posterior distributions for individual heterogeneity in movement probability, or sample variance of mean probabilities of movement among individual brown treesnakes by treatment group when a snake was located in each listed landscape feature var($\mu _{p_{t}}$). Resident snakes were non-translocated snakes in an urban area, forest to urban snakes were translocated from a forest to an urban area, and urban to urban snakes were translocated from an urban to a novel urban area. **b** Heat map of marginal posterior mean difference in individual heterogeneity, or the sample variance of individual brown treesnake movement probabilities between treatment groups. The colors represent the difference in the first listed treatment group’s posterior mean compared to the second listed treatment group’s posterior mean. The posterior probability that the first listed treatment group is greater than the second listed treatment group is printed in each cell

Resident snakes had the largest posterior mean sample variances among individual estimates (Fig. 2a), but differences in this measure of individual heterogeneity among treatment groups were overall small (Fig. 2b). The largest difference in individual heterogeneity occurred when snakes were on grass: Resident snakes had a movement probability sample variance 0.05 greater than snakes translocated from forests with posterior probability of 0.95 (Fig. 2b).

**Fig. 3:**
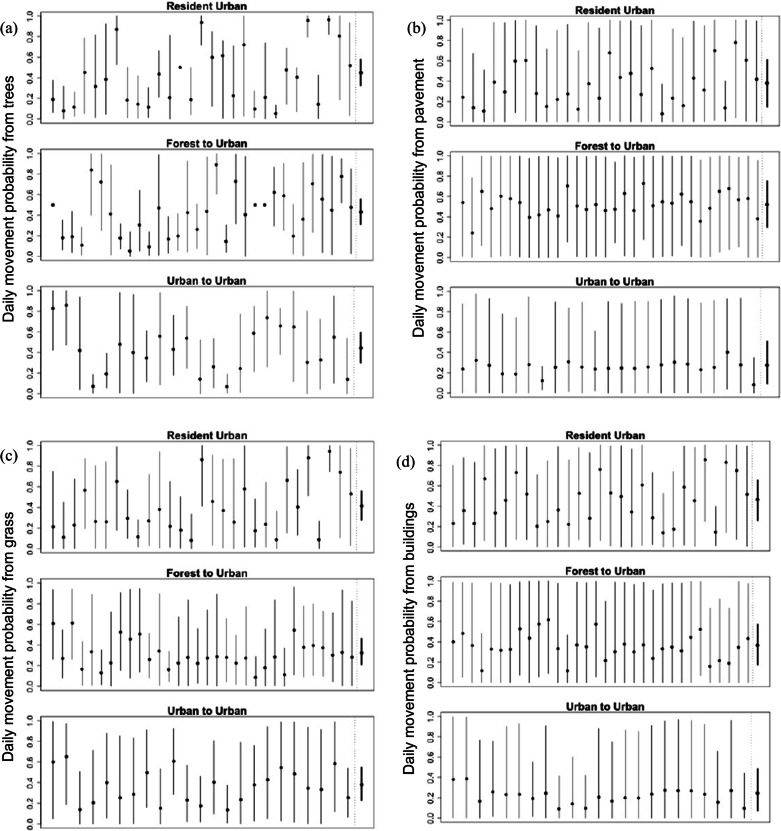
Posterior means and 95% credible intervals for individual brown treesnake (thin lines, $p_{it}$) and treatment-level (bold lines, $\mu _{p_{t}}$) probability of movement when a snake is located in or on **a** trees, **b** pavement, **c** grass, and **d** buildings. Resident snakes were non-translocated snakes in an urban area, forest to urban snakes were translocated from a forest to an urban area, and urban to urban snakes were translocated from an urban area to a novel urban area

Some individuals had particularly high movement probabilities across all landscape features. Namely, resident snakes #7, #15, #25, #27, and #28 had posterior mean movement probabilities >0.60 on all landscape features and >0.80 in trees, while treatment-level posterior means for resident snakes were <0.50 in all small-landscape features (posterior means 0.45 in buildings, 0.38 in grass, 0.41 on pavement, and 0.47 in trees, Fig. 3). This suggests some snakes may have a higher propensity for movement, regardless of their environment; however, the uncertainty in individual estimates limits our understanding of differences among individuals in this study.

### Movement state parameters

**Fig. 4:**
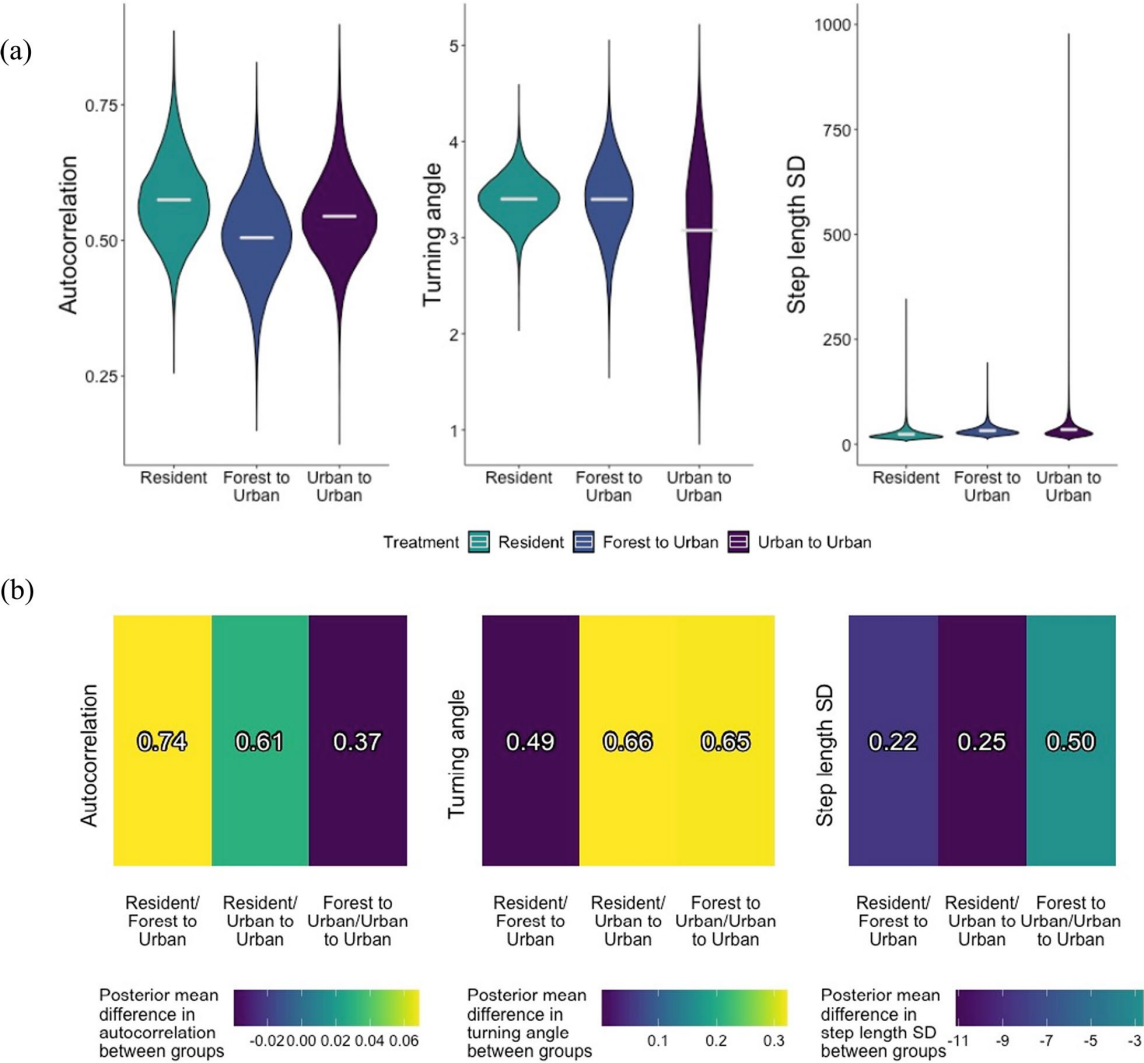
**a** Marginal posterior distributions for treatment-level autocorrelation $\mu _\gamma$, turning angle $\mu _\theta$, and step length standard deviation $\mu _\sigma$ parameters dictating the brown treesnake movement process. Resident snakes were non-translocated snakes in an urban area, forest to urban snakes were translocated from a forest to an urban area, and urban to urban snakes were translocated from an urban to a novel urban area. **b** Heat maps of marginal posterior mean differences in brown treesnake autocorrelation, turning angle, and step length standard deviation parameters between treatment groups. The colors represent the difference in the first listed treatment group’s posterior mean compared to the second listed treatment group’s posterior mean. The posterior probability that the first listed treatment group is greater than the second listed treatment group is printed in each cell

Posterior mean step length parameters $\mu _{\sigma _1}$ ranged from 23 to 34 m (Fig. 4a), which translates to 95% movement kernels of 46 and 68 m each night. We found weak evidence that translocated brown treesnakes made longer nightly movements when in the movement state than resident snakes. Snakes translocated from forests had a posterior mean step length parameter 35% greater than residents, and for snakes translocated from urban areas, this parameter was 47% greater than resident snakes. However, these estimates had high uncertainty, lessening the strength of this effect (respective posterior probabilities 0.22 and 0.25, Fig. 4b).

**Fig. 5:**
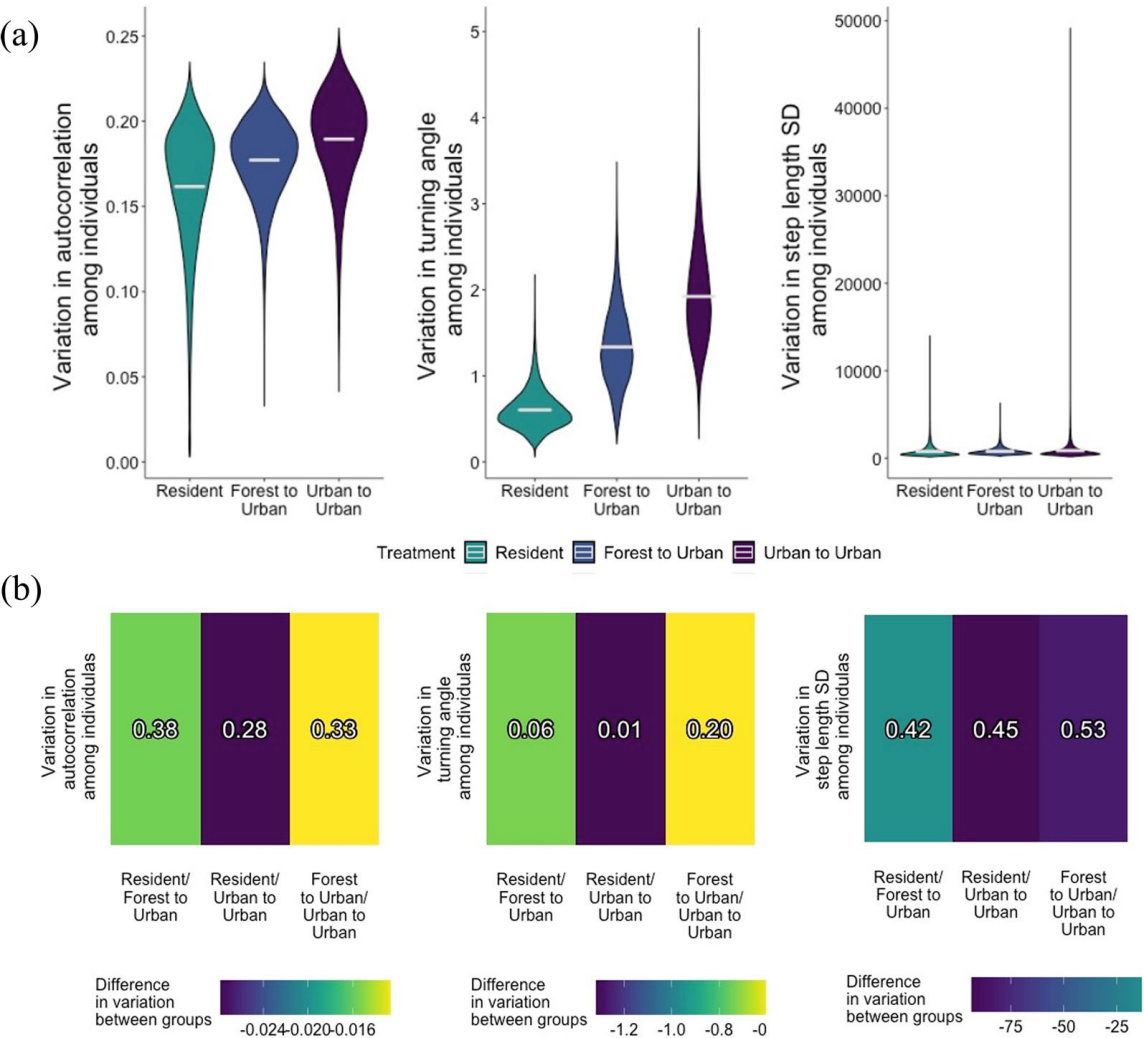
**a** Marginal posterior distributions for treatment-level individual heterogeneity in movement parameters, or sample variance in autocorrelation var($\mu _\gamma$), turning angle var($\mu _\theta$), and step length standard deviation var($\mu _\sigma$) parameters among individual brown treesnakes. Resident snakes were non-translocated snakes in an urban area, forest to urban snakes were translocated from a forest to an urban area, and urban to urban snakes were translocated from an urban to a novel urban area. **b** Heat maps of marginal posterior mean differences in brown treesnake individual heterogeneity, or sample variance in autocorrelation, turning angle, and step length standard deviation parameters between treatment groups. The colors represent the difference in the first listed treatment group’s posterior mean compared to the second listed treatment group’s posterior mean. The posterior probability that the first listed treatment group is greater than the second listed treatment group is printed in each cell

**Fig. 6:**
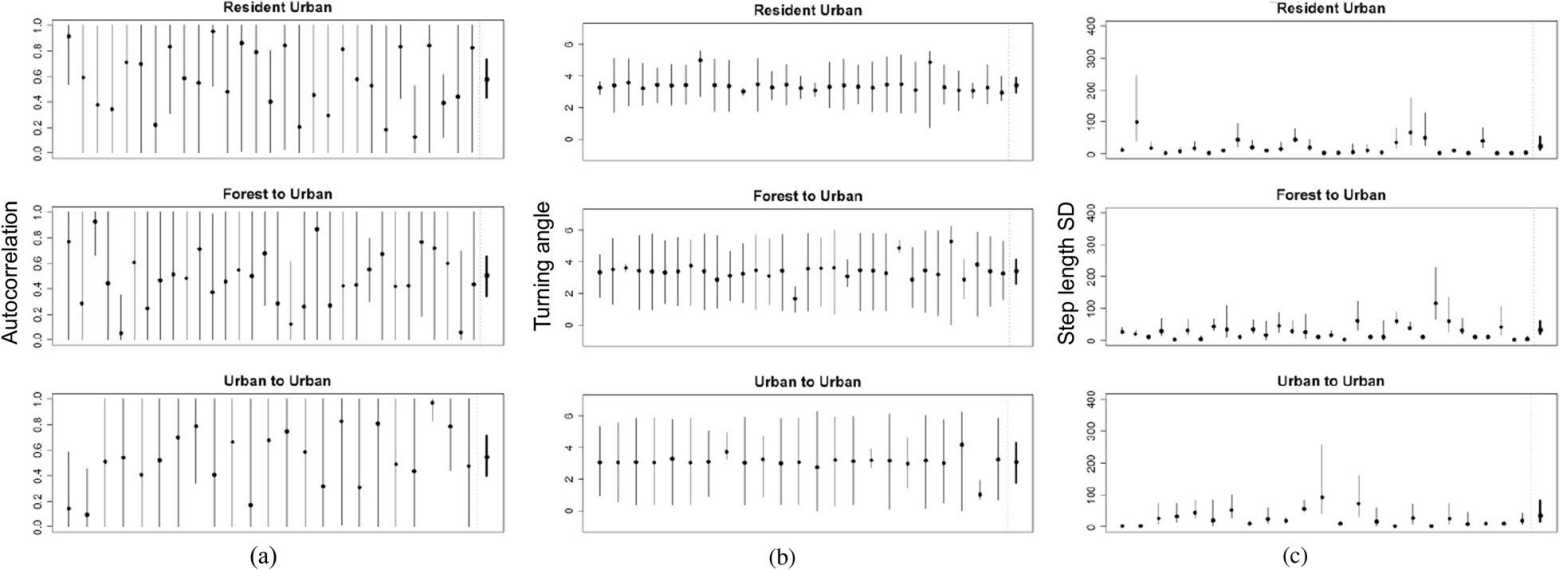
Posterior means and 95% credible intervals for individual brown treesnake (thin lines) and treatment-level (bold lines) **a** autocorrelation $\gamma$, **b** turning angle $\theta$, and **c** step length standad $\sigma _1$ parameters controlling the movement process. Resident snakes were non-translocated snakes in an urban area, forest to urban snakes were translocated from a forest to an urban area, and urban to urban snakes were translocated from an urban to a novel urban area

There were no strong differences in individual heterogeneity in step length among treatment groups (Fig. 5), however individual heterogeneity was high overall for this parameter. Some individual $\sigma _{1,i}$ posterior mean estimates were close to 100 (resident #2, forest to urban #25, urban to urban #12, Fig. 6c), which corresponds to a nightly 95% movement kernel of >200 m.

All snakes’ posterior means for treatment-level $\mu _\theta$ were close to $\pi$, indicating that snakes generally moved in the opposite direction ($180^{\circ }$) of their last movement, (i.e., doubled-back on their trajectory; Fig. 4a). We found no strong differences in turning angle among treatment groups, with posterior probabilities close to 0.50 (Fig. 4b).

Individual heterogeneity in $\theta _i$ was low, with most snakes’ individual estimates close to $\pi$ (Fig. 6b). Resident snakes had the lowest individual heterogeneity in turning angles, with posterior mean sample variance estimates 0.73 and 1.3 lower than translocated snakes from forests and urban areas, respectively (posterior probabilities 0.06 and 0.01, Fig. 5b). Forest to urban snakes had a posterior mean sample variance in turning angles 0.59 lower than urban to urban snakes, but this difference was weak (posterior probability 0.20, Fig. 5b). This suggests translocated snakes make a higher diversity of turning angles among individuals than resident snakes, but without a strong effect of habitat of origin.

The autoregression parameter $\gamma$ affects our interpretation of $\theta$, however, because it determines how closely an individual’s turning angles adhere to $\theta$ at each step. Posterior mean estimates of $\mu _\gamma$ ranged from 0.51 to 0.57 among the treatment groups, values that relax the trajectories’ adherence to $\theta$ and creates more natural (or less patterned) movement paths (Fig. 4a). We found no strong differences in autoregression parameter estimates among the three treatment groups (Fig. 4b). Specifically, we learned that snakes in all treatment groups tended to make about-face turns because $\mu _{\theta }$ was estimated near $\pi$, but snakes did not make about-face turns at every step, because $\mu _\gamma<$1.

Individual heterogeneity in $\gamma _i$ was relatively high, with individual posterior mean estimates ranging from 0.05 to 0.97, however 95% credible intervals for individual $\gamma _i$ estimates were large (Fig. 6a), indicating high uncertainty in these estimates. There were no strong differences in sample variance in individual-level autoregression parameters among the three treatment groups (Fig. 5b).

## Discussion

Our ability to prevent the spread of invasive species depends upon our understanding of the mechanistic components of a species’ movements through the landscapes it invades. The ecology of invasive reptiles creates unique challenges to modeling their movement, including sporadic movements and individual heterogeneity in behavior. We developed a Bayesian hierarchical model to accommodate these factors and to answer questions about how an invasive reptile’s movement mechanisms are affected by translocation. We also aimed to understand how small-scale landscape features and habitat of origin affect brown treesnake movement. Though mechanistic modeling has been used to analyze movements of a variety of taxa, our work is the first to gain this type of mechanistic inference on invasive reptile movement.

Our findings highlight the importance of accounting for individual heterogeneity when researching and managing invasive species. We did not find strong, consistent effects of translocation or habitat origin on brown treesnake movement, mostly due to variation among individuals within treatment groups. Variation could be reduced by increasing the number of snakes tracked, but our sample size is comparatively high for cryptic reptiles that must be tracked manually to gain accurate relocation data. By accounting for individual heterogeneity within treatment groups, we can infer the consistency of treatment effects among individuals. For example, we found weak evidence for resident snakes to be the least consistent in their response to small-scale landscape features, while translocated snakes from urban areas generally behaved more similarly to one another across landscape features, especially on buildings and pavement. This suggests translocation could create relatively consistent changes in snake movement behavior. Additionally, it highlights the baseline variation in snakes residing in familiar urban areas.

We found evidence that some brown treesnakes respond to novel small-scale landscape features by increasing their movement probability. Translocated snakes inhabiting small-scale landscape features less common to their habitat of origin moved more often than resident snakes. Specifically, snakes translocated from urban areas moved out of trees more than from buildings, while snakes translocated from forests moved off pavement more than grass. This suggests that, in a rapid response scenario, determining the most likely origin habitat of a translocated individual can inform how quickly it disperses through a novel landscape. For example, brown treesnakes accidentally translocated via Guam’s transit system may be considered “urban” snakes and could disperse more quickly across other, more forested, islands in the Commonwealth of the Northern Mariana Islands. The lack of a consistent, generalized effect of translocation on movement probability agrees with studies in temperate environments that found no difference in movement frequency, a proxy for movement probability, between resident and translocated snakes [44–46], suggesting its effects are context-specific.

This landscape context is also important to consider when managing snakes in different environments (i.e., forest vs. urban). Previous research has found brown treesnakes avoid roads [64], however, road crossings in our urban study area were nearly unavoidable for a species with an estimated activity area of 20 ha [65]. Although multiple individuals crossed roads, increased movement probabilities around pavement suggest brown treesnakes translocated from forests avoid using paved surfaces as refugia for multiple days, which is consistent with previous research. Our inferred differences among treatment groups were weak, but we found that snakes translocated from urban areas had the lowest estimates of movement probability when located on buildings and pavement. This suggests that how a translocated individual disperses through a novel environment is both dependent on its habitat of origin and the new environment it encounters. However, more research is needed to determine if these snakes preferentially select these urban landscape features that are similar to their habitat of origin.

Our observed individual heterogeneity in movement patterns could be attributed to internal factors we did not model, such as size or sex. However, we did not expect large differences in movement based on size. Brown treesnakes sexually mature when they reach 910–1025 mm snout-to-vent length for females and 940-1,030 mm for males [66]. Because only five of our snakes were >900 mm snout-to-vent length, we did not include sex or reproductive status in our model. Additionally, post-hoc Pearson’s correlations (Additional File 4: Table S1) and comparisons of individual-level posterior mean parameter estimates (Additional File 4: Figs. S1 and S2) did not show strong relationships between movement parameter estimates and snake sex or size.

New invasions of translocated brown treesnakes may spread faster over an urban landscape than what we would expect in a resident population. We found weak evidence that translocated brown treesnakes made longer movements and strong evidence for differences in variation in turning angles among translocated individuals compared to resident snakes. These findings generally agree with previous translocation research on other snake species that found longer [44, 46, 49, 67] and more unidirectional daily movements in translocated snakes [44, 45, 50, 68]. Again, individual heterogeneity is evident in our results, because we did not find treatment-level differences in baseline turning angles, but all treatment group posterior means centered around 180$^{\circ }$ and translocated snakes had more individual heterogeneity around this mean, suggesting they make more directed movements than residents. The relatively weak effect of treatment group would suggest that surveillance efforts may not need to consider whether a snake was a recent introduction when designing search protocols, however a cautious approach may be to use parameter estimates at the tail of the estimated distributions in movement simulations to ensure coverage of dispersing individuals.

## Conclusions

Our work contributes to understanding the mechanisms of invasive reptile movement and how these movements are affected by endogenous and exogenous factors to better manage one of the most destructive invasive species to date. Our analysis accounts for the aspects of reptile ecology that make this group especially difficult to study and manage: Their sporadic movements and individual heterogeneity. Our approach also provides a starting point for early detection and rapid response planning, allowing managers to visualize a range of potential invasion scenarios.

Our model is not limited to analyzing nightly movements at small spatial scales. It can be applied to taxa that move much farther distances, as long as landscape covariate data are available at a scale appropriate to the animal’s movements. Discrete-time models, like the one we present here, are best suited for data with low temporal resolution, and are appropriate for analyzing telemetry data from animals tracked no more than once daily. Data at finer temporal resolutions can be analyzed using continuous-time movement models, which mimic the natural movement process [69].

Our modeling approach can also be generalized to accommodate different forms of error and variation. In our study of brown treesnakes, we collected data that yielded accurate measurements of individual position, but in other systems, methods exist to account for unusual forms of measurement error that may arise in both radio [52] or satellite [70, 71] telemetry studies. Alternatively, with more data, another hierarchy could be added to account for seasonal variation. Guam’s wet season tends to be warmer than its dry season, and one study found an increase in brown treesnake movement probability during the wet season, but the effect was weak [64]. Our study did not include enough snakes per treatment group per season to analyze differences in seasonal variation, and our estimates of uncertainty accommodate seasonal variation. Due to Guam’s relatively stable tropical climate, we did not expect the difference in mechanistic movement parameters between seasons to be large enough to warrant another level of hierarchy in the model.

Our approach to modeling movement has broad applications for invasive species ecology and management. For example, Burmese pythons have been spreading through the Florida Keys, USA since 2002, but as of 2018 have not spread to the southernmost keys [72]. Our model could be fit to existing python movement data in Florida and the resulting movement parameters could be used to determine how quickly the invasion front is likely to expand. Also in the southeastern United States, Argentine black and white tegus (*Salvator merianae*) are now established in Florida and Georgia from captive releases with indications of spread from those release sites [73]. When fit to tegu movement data, our model can be used to predict where and how quickly tegus are likely to spread from reported sighting locations. Our approach can account for seasonal variations in behavior that occur in temperate climates, such as tegu brumation [74–76], by specifying movement probability as a function of temperature. In both of these applications, managers can obtain more biologically informed estimates of the spatial extent of an invasion as well as an understanding of invasion speed, helping them decide when and where they should direct interdiction or rapid response efforts.

## Supplementary Information

**Additional file 1.** Full model specification.

**Additional file 2.** Second stage Metropolis-Hastings ratios.

**Additional file 3.** Snakes moving to contiguous forest.

**Additional file 4.** Differences in size and sex among snakes.

## Data Availability

The datasets generated and/or analyzed during the current study are available as a USGS data release, 10.5066/P948KRN3 [77]. Code to fit the model and create figures for this manuscript is available at https://code.usgs.gov/fort/ecosystems-reptile_movement_model.

## Abbreviations

CI: Credible interval
MH: Metropolis-Hastings
VHF: Very high frequency
