# Supplementary material for: Individual heterogeneity influences the effects of translocation on urban dispersal of an invasive reptile
Source: Mov Ecol. 2022 Jan 15;10:2. doi: 10.1186/s40462-022-00300-1 (PMC8761355; doi:10.1186/s40462-022-00300-1)
Supplement: Supplementary file 1 — Additional file 1. Full model specification. [file 40462_2022_300_MOESM1_ESM.pdf]

Feuka, A. B., Nafus, M. G., Yackel Adams, A. A., Bailey, L. L., and Hooten, M. B. 2022. Individual heterogeneity influences the effects of translocation on urban dispersal of an invasive reptile. *Movement Ecology*.

Additional File 1 - Full model specification

We specified the full movement model we fit to brown treesnake relocation data as

$$
\begin{aligned}
\boldsymbol{\delta}_{it} &= \mathbf{s}_{it} - \mathbf{s}_{it-1}, \quad \text{for } t = 3, ..., \mathrm{T}_i \text{ and } i = 1, ..., \mathrm{N}_j, \\
\boldsymbol{\delta}_{it} &\sim \begin{cases} \mathrm{N}(\gamma_i \mathbf{M}(\theta_i)\boldsymbol{\delta}_{it-1}, \sigma^2_{1,i}\mathbf{I}), & \text{with probability } p_{it}, \\ \mathrm{N}(\mathbf{0}, \sigma^{\mathbf{2}}_{\mathbf{0}}\mathbf{I}), & \text{with probability } 1 - p_{it}, \end{cases} \\
p_{it} &= \Phi(\boldsymbol{x}'_{it-1}\boldsymbol{\beta}_i), \\
\boldsymbol{\beta}_i &\sim \mathrm{N}(\boldsymbol{\mu}_{\beta,j}, \boldsymbol{\Sigma}_{\beta,j}), \quad \text{for } j = 1, ..., 3, \\
\text{logit}(\gamma_i) &\sim \mathrm{N}(\mu_{\text{logit}(\gamma),j}, \sigma^2_{\text{logit}(\gamma),j}), \\
\theta_i &\sim \mathrm{N}(\mu_{\theta,j}, \sigma^2_{\theta,j}), \\
\log(\sigma_{1,i}) &\sim \mathrm{N}(\mu_{\log(\sigma_1),j}, \sigma^2_{\log(\sigma_1),j}), \\
\boldsymbol{\mu}_{\beta,j} &\sim \mathrm{N}(\boldsymbol{\mu}_{\beta,\text{pop}}, \boldsymbol{\Sigma}_{\beta,\text{pop}}), \\
\mu_{\text{logit}(\gamma),j} &\sim \mathrm{N}(\mu_{\text{logit}(\gamma),\text{pop}}, \sigma^2_{\text{logit}(\gamma),\text{pop}}), \\
\mu_{\theta,j} &\sim \mathrm{N}(\mu_{\theta,\text{pop}}, \sigma^2_{\theta,\text{pop}}), \\
\mu_{\log(\sigma_{1,j})} &\sim \mathrm{N}(\mu_{\log(\sigma_1),\text{pop}}, \sigma^2_{\log(\sigma_1),\text{pop}}), \\
\boldsymbol{\Sigma}^{-1}_{\beta,j} &\sim \text{Wish}((\mathbf{S}\nu)^{-1}, \nu), \\
\sigma^2_{\text{logit}(\gamma),j} &\sim \text{IG}(q_{\text{logit}(\gamma),\text{pop}}, r_{\text{logit}(\gamma),\text{pop}}), \\
\sigma^2_{\theta,j} &\sim \text{IG}(q_{\theta,\text{pop}}, r_{\theta,\text{pop}}), \\
\sigma^2_{\log(\sigma_{1,j})} &\sim \text{IG}(q_{\log(\sigma_1),\text{pop}}, r_{\log(\sigma_1),\text{pop}}),
\end{aligned}
\qquad \text{(Eqn S1)}
$$

where $\mathbf{s}_{it}$ indicates the position of individual i on day t. When a snake was in the movement state, its movement at time t, $\boldsymbol{\delta}_{it}$, was auto-regressed on its last movement $\boldsymbol{\delta}_{it-1}$. This previous night's movement was multiplied by the propagation matrix $\mathbf{M}$, which rotates the direction of $\boldsymbol{\delta}_{it}$ using

$$\mathbf{M} \equiv \begin{pmatrix} \cos(\theta_i) & -\sin(\theta_i) \\ \sin(\theta_i) & \cos(\theta_i) \end{pmatrix}, \qquad \text{(Eqn S2)}$$

in which θ_i controls the turning angle (in radians) of snake i. To create more realistic trajectories, we multiplied the autoregression coefficient γ_i to each snake's propagation matrix to dampen its effect. The variance parameters, σ_0^2 and $\sigma_{1,i}^2$ multiplied by identity matrix $\mathbf{I}$, control the variation in step length and direction for each state, where twice the standard deviation accounts for 95% of the movement kernel at each step. We fixed $\sigma_0^2 = 25$ based on the estimated amount of movement in an encamped state. We specified movement probability p_{it} as a probit-linear function of the small-scale landscape feature a snake was using at the starting position of each movement, $\boldsymbol{x}_{it-1}$, specifically trees, grass, pavement, or buildings.

Each individual snake had its own set of movement parameters (γ_i, θ_i, and $\sigma_{1,i}^2$) and parameters dictating the probability of movement ($\boldsymbol{\beta}_i$). Our individual-level process model was comprised of these parameters, which arose from normal distributions that are parameterized by treatment-level means and variances, indexed by j. Experimental treatments groups in this study included resident snakes, snakes translocated from forests, and snakes translocated from urban areas. We used treatment-level means to determine differences in movement patterns among treatment groups and we used treatment-level variance parameters to quantify individual heterogeneity in movement patterns within each group.
