# Supplementary material for: Individual heterogeneity influences the effects of translocation on urban dispersal of an invasive reptile
Source: Mov Ecol. 2022 Jan 15;10:2. doi: 10.1186/s40462-022-00300-1 (PMC8761355; doi:10.1186/s40462-022-00300-1)
Supplement: Supplementary file 2 — Additional file 2. Second stage Metropolis-Hastings ratios. [file 40462_2022_300_MOESM2_ESM.pdf]

Feuka, A. B., Nafus, M. G., Yackel Adams, A. A., Bailey, L. L., and Hooten, M. B. 2022. Individual heterogeneity influences the effects of translocation on urban dispersal of an invasive reptile. *Movement Ecology*.

Additional File 2 - Second Stage Metropolis-Hastings Ratios

In the second stage of model fitting, we used the following generalized Metropolis-Hastings ratio:

$$r_{jl}^{k} = \frac{\left[\boldsymbol{y}_i|\alpha_{il}^{*}, \alpha_{i,1:l-1}^{k}, \alpha_{i,l+1:L}^{k-1}\right]\left[\alpha_{il}^{*}|\boldsymbol{\psi}, \alpha_{i,1:l-1}^{k}, \alpha_{i,l+1:L}^{k-1}\right]\left[\alpha_{il}^{k-1}|\mathbf{y}_i\right]_{*}}{\left[\boldsymbol{y}_i|\alpha_{il}^{k-1}, \alpha_{i,1:l-1}^{k}, \alpha_{i,l+1:L}^{k-1}\right]\left[\alpha_{il}^{k-1}|\boldsymbol{\psi}, \alpha_{i,1:l-1}^{k}, \alpha_{i,l+1:L}^{k-1}\right]\left[\alpha_{il}^{*}|\mathbf{y}_i\right]_{*}}. \qquad \text{(Eqn S1)}$$

where α_{il} is the lth parameter in the vector of parameters for individual i. Superscript k indicates the index for the current MCMC iteration, $*$ indicates a proposed value, $\boldsymbol{\psi}$ is a vector of hyperparameters for α_{il}, and $_{*}$ indicates the second-stage proposal distribution (not the second-stage marginal posterior distribution of α_{il}). Here, $\left[\boldsymbol{y}_i|\alpha_{il}^{*}, \alpha_{i,1:l-1}^{k}, \alpha_{i,l+1:L}^{k-1}\right]$ is the likelihood of the data given the individual-level model parameters, one of which is being updated (α_{il}^{*}), some that have already been updated ($\alpha_{i,1:l-1}^{k}$), and some that remain to be updated ($\alpha_{i,l+1:L}^{k-1}$) in MCMC iteration k.

The process distribution for α_{il}^{*}, $\left[\alpha_{il}^{*}|\boldsymbol{\psi}, \alpha_{i,1:l-1}^{k}, \alpha_{i,l+1:L}^{k-1}\right]$, accounts for cases in which α_{il}^{*} is correlated with other parameters in the second stage. This is the case for $\boldsymbol{\beta}_i$ in our model, which were sampled jointly in the first stage and individually in the second stage, but remain correlated with one another in the second stage. Therefore, when updating each β_{il} in the second stage, the prior uses the joint distribution

$$\begin{pmatrix} \boldsymbol{\beta}_o \\ \boldsymbol{\beta}_u \end{pmatrix} \sim \mathrm{N}\left(\begin{pmatrix} \boldsymbol{\mu}_{\boldsymbol{\beta},o} \\ \boldsymbol{\mu}_{\boldsymbol{\beta},u} \end{pmatrix}, \begin{pmatrix} \boldsymbol{\Sigma}_{\boldsymbol{\beta},oo}, \boldsymbol{\Sigma}_{\boldsymbol{\beta},ou} \\ \boldsymbol{\Sigma}_{\boldsymbol{\beta},uo}\boldsymbol{\Sigma}_{\boldsymbol{\beta},uu} \end{pmatrix}\right) \qquad \text{(Eqn S2)}$$

to account for the fact that some β coefficients have been updated, and some have not (Cressie, 1990; Hooten, Johnson, McClintock, et al., 2017). Therefore, the process distribution for each β_{il} is $\left[\beta_{il}|\boldsymbol{\mu}_{\beta,j}, \boldsymbol{\Sigma}_{\beta,j}, \beta_{i,1:l-1}^{k}, \beta_{i,l+1:L}^{k-1}\right]$.

In two-stage proposal-recursive computation, the proposal distribution is the marginal posterior distribution from the first stage $[\alpha_{il}|\mathbf{y}_i]_*$, where $_*$ denotes this as the second-stage proposal distribution and not the second-stage posterior distribution of α_{il}. To reduce computation time, we approximated $[\alpha_{il}|\mathbf{y}_i]_*$ using the kernel density estimate calculated from each parameter's first-stage samples. We thus obtain the following MH ratio for each β_{il}:

$$r_{jl}^{k} = \frac{\begin{gathered}\left(\prod_{t=3}^{T_i}\left[\boldsymbol{\delta}_{it}|\gamma_i^k, \theta_i^k, \sigma_{1,i}^{2(k)}, \beta_{il}^*, \beta_{i,1:l-1}^k, \beta_{i,l+1:L}^{k-1}\right]\right) \times \\ \left[\beta_{il}^*|\boldsymbol{\mu}_{\beta,j}, \boldsymbol{\Sigma}_{\beta,j}, \beta_{i,1:l-1}^k, \beta_{i,l+1:L}^{k-1}\right]\left[\beta_{il}^{k-1}|\boldsymbol{\delta}_i\right]_*\end{gathered}}{\begin{gathered}\left(\prod_{t=3}^{T_i}\left[\boldsymbol{\delta}_{it}|\gamma_i^k, \theta_i^k, \sigma_{1,i}^{2(k)}, \beta_{il}^{k-1}, \beta_{i,1:l-1}^k, \beta_{i,l+1:L}^{k-1}\right]\right) \times \\ \left[\beta_{il}^{k-1}|\boldsymbol{\mu}_{\beta,j}, \boldsymbol{\Sigma}_{\beta,j}, \beta_{i,1:l-1}^k, \beta_{i,l+1:L}^{k-1}\right]\left[\beta_{il}^*|\boldsymbol{\delta}_i\right]_*\end{gathered}}. \quad \text{(Eqn S3)}$$

For movement parameters γ_i, θ_i, and $\sigma_{1,i}^2$ in our model, the process distribution is simplified because these parameters are sampled individually in the second stage. The generalized process distribution for these parameters simplifies to $[\alpha_{il}|\boldsymbol{\psi}]$, where $\boldsymbol{\psi}$ are parameters from each movement parameter's prior distribution. This results in the following MH ratio for γ_i, θ_i, and $\sigma_{1,i}^2$, simplified from S1:

$$r_{jl}^{k} = \frac{\left[\boldsymbol{y}_i|\alpha_{il}^*, \alpha_{i,1:l-1}^k, \alpha_{i,l+1:L}^{k-1}\right]\left[\alpha_{il}^*|\boldsymbol{\psi}\right]\left[\alpha_{il}^{k-1}|\mathbf{y}_i\right]_*}{\left[\boldsymbol{y}_i|\alpha_{il}^{k-1}, \alpha_{i,1:l-1}^k, \alpha_{i,l+1:L}^{k-1}\right]\left[\alpha_{il}^{k-1}|\boldsymbol{\psi}\right]\left[\alpha_{il}^*|\mathbf{y}_i\right]_*}. \quad \text{(Eqn S4)}$$
