# Supplementary material for: Individual heterogeneity influences the effects of translocation on urban dispersal of an invasive reptile
Source: Mov Ecol. 2022 Jan 15;10:2. doi: 10.1186/s40462-022-00300-1 (PMC8761355; doi:10.1186/s40462-022-00300-1)
Supplement: Supplementary file 3 — Additional file 3. Snakes moving to contiguous forest. [file 40462_2022_300_MOESM3_ESM.pdf]

Feuka, A. B., Nafus, M. G., Yackel Adams, A. A., Bailey, L. L., and Hooten, M. B. 2022. Individual heterogeneity influences the effects of translocation on urban dispersal of an invasive reptile. *Movement Ecology*.

Additional File 3 - Snakes moving to contiguous forest

Table S1: Information on brown treesnakes that moved outside of our urban study site into surrounding contiguous forest. For each of these snakes we report their first day in the study, the day they reached the forest, how long it took them to reach the forest, and the Euclidean distance traveled (in meters) between the two dates. Resident snakes (R) were non-translocated snakes in an urban area, forest to urban snakes (FU) were translocated from a forest to an urban area, and urban to urban snakes (UU) were translocated from an urban to a novel urban area.

Snake ID	Treatment	Start Date	Date Forest Reached	Days to Forest	Euclidean distance
4007	R	06/25/18	07/11/18	16	104
4011	FU	07/08/18	08/13/18	36	258
4021	FU	07/17/18	08/29/18	43	190
4047	R	02/21/19	03/31/19	38	97
4051	UU	02/27/19	03/03/19	4	267
4056	FU	03/06/19	03/18/19	12	282
4060	FU	03/07/19	04/06/19	30	259
4073	R	03/09/19	03/10/19	1	67
4078	UU	03/15/19	03/20/19	5	281
4612	FU	07/19/19	08/02/19	14	243
