# Supplementary material for: Individual heterogeneity influences the effects of translocation on urban dispersal of an invasive reptile
Source: Mov Ecol. 2022 Jan 15;10:2. doi: 10.1186/s40462-022-00300-1 (PMC8761355; doi:10.1186/s40462-022-00300-1)
Supplement: Supplementary file 4 — Additional file 4. Differences in size and sex among snakes. [file 40462_2022_300_MOESM4_ESM.pdf]

Feuka, A. B., Nafus, M. G., Yackel Adams, A. A., Bailey, L. L., and Hooten, M. B. 2022. Individual heterogeneity influences the effects of translocation on urban dispersal of an invasive reptile. *Movement Ecology*.

Additional File 4 - Differences in size and sex among snakes

Table S1: Pearson's correlations between individual-level movement parameter posterior means estimates and brown treesnake snout-to-vent length (in mm) for each experimental treatment group. Resident snakes were non-translocated snakes in an urban area, forest to urban snakes were translocated from a forest to an urban area, and urban to urban snakes were translocated from an urban to a novel urban area.

	Resident	Forest to Urban	Urban to Urban
γ_i	-0.20	-0.07	-0.13
θ_i	0.08	-0.18	-0.15
σ_i	-0.01	0.14	-0.10
$p_{it,tree}$	0.16	0.29	0.19
$p_{it,pavement}$	0.19	0.18	-0.03
$p_{it,grass}$	0.25	-0.20	-0.01
$p_{it,building}$	0.17	0.06	-0.11

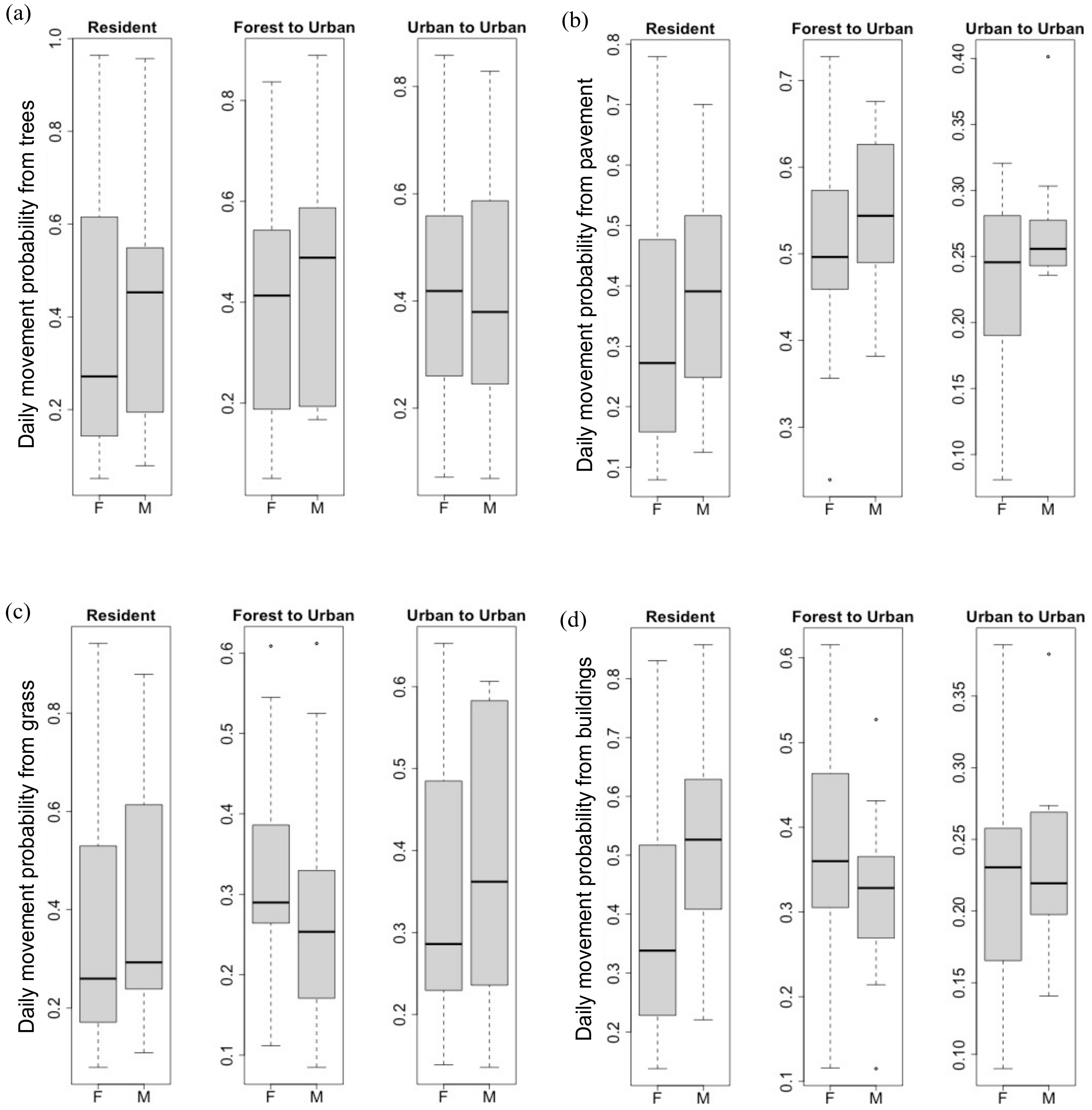

Figure S1: Comparison of individual-level posterior mean estimates of movement probabilities μ_{p_t} between male and female snakes when located in or on (a) trees, (b) pavement, (c) grass, and (d) buildings between sexes. Resident snakes were non-translocated snakes in an urban area, forest to urban snakes were translocated from a forest to an urban area, and urban to urban snakes were translocated from an urban to a novel urban area. Box plots depict the minimum, first quartile, median, third quartile, and maximum, with outliers depicted as single points.

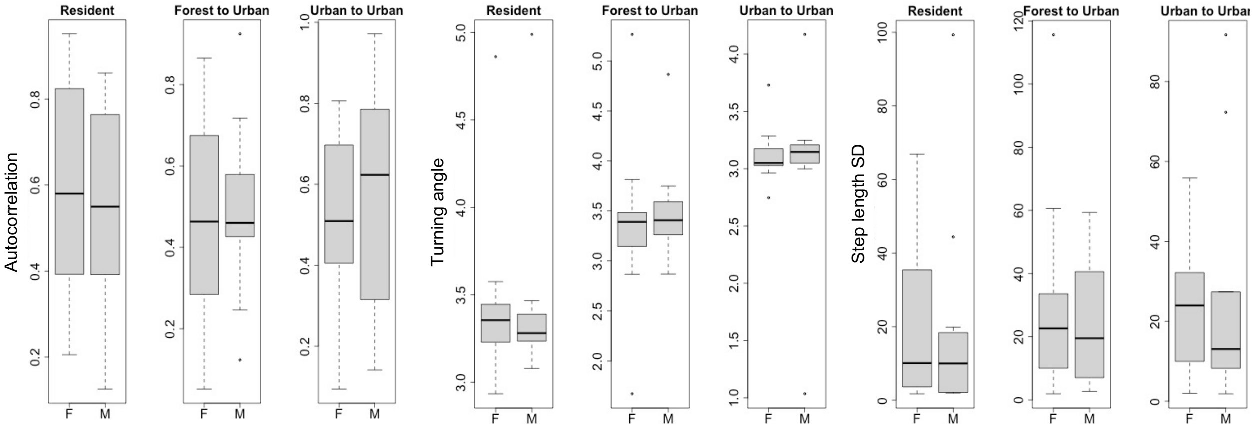

(a) (b) (c)

Figure S2: Comparison of individual-level posterior mean estimates between male and female snakes for (a) autocorrelation γ, (b) turning angle θ, and (c) step length σ_1 parameters. Resident snakes were non-translocated snakes in an urban area, forest to urban snakes were translocated from a forest to an urban area, and urban to urban snakes were translocated from an urban to a novel urban area. Box plots depict the minimum, first quartile, median, third quartile, and maximum, with outliers depicted as single points.
